# Social Vulnerability and Ebola Virus Disease in Rural Liberia

**DOI:** 10.1371/journal.pone.0137208

**Published:** 2015-09-01

**Authors:** John A. Stanturf, Scott L. Goodrick, Melvin L. Warren, Susan Charnley, Christie M. Stegall

**Affiliations:** 1 Center for Forest Disturbance Science, U.S. Forest Service, Athens, Georgia, United States of America; 2 Center for Bottomland Hardwoods Research, U.S. Forest Service, Oxford, Mississippi, United States of America; 3 Goods, Services and Values Program, U.S. Forest Service, Portland, Oregon, United States of America; INDEPTH Network, GHANA

## Abstract

The Ebola virus disease (EVD) epidemic that has stricken thousands of people in the three West African countries of Liberia, Sierra Leone, and Guinea highlights the lack of adaptive capacity in post-conflict countries. The scarcity of health services in particular renders these populations vulnerable to multiple interacting stressors including food insecurity, climate change, and the cascading effects of disease epidemics such as EVD. However, the spatial distribution of vulnerable rural populations and the individual stressors contributing to their vulnerability are unknown. We developed a Social Vulnerability Classification using census indicators and mapped it at the district scale for Liberia. According to the Classification, we estimate that districts having the highest social vulnerability lie in the north and west of Liberia in Lofa, Bong, Grand Cape Mount, and Bomi Counties. Three of these counties together with the capital Monrovia and surrounding Montserrado and Margibi counties experienced the highest levels of EVD infections in Liberia. Vulnerability has multiple dimensions and a classification developed from multiple variables provides a more holistic view of vulnerability than single indicators such as food insecurity or scarcity of health care facilities. Few rural Liberians are food secure and many cannot reach a medical clinic in <80 minutes. Our results illustrate how census and household survey data, when displayed spatially at a sub-county level, may help highlight the location of the most vulnerable households and populations. Our results can be used to identify vulnerability hotspots where development strategies and allocation of resources to address the underlying causes of vulnerability in Liberia may be warranted. We demonstrate how social vulnerability index approaches can be applied in the context of disease outbreaks, and our methods are relevant elsewhere.

## Introduction

The recent Ebola virus disease (EVD) outbreak in West Africa first appeared in Guinea in December 2013, where it is thought to have been initially transmitted to a two-year old boy playing in a hollow tree that harbored a colony of insectivorous free-tailed bats (*Mops condylurus*) [1]. The first case in Liberia was reported in Lofa County (on the border with Guinea) in March 2014 [2]. By July 2014, cases were reported in the Liberian capital Monrovia. Of the West African countries where EVD has since occurred, Liberia had the second highest number of cumulative confirmed, probable, and suspected cases between mid- August 2014 and May 2015 [3]. The World Health Organization proclaimed Liberia to be Ebola free as of May 9, 2015; but it had suffered 10,604 cases (40% of the total), and 4,769 deaths, 43% of the mortality in West Africa as of May 10, 2015 [3]. These statistics put Liberia just behind Sierra Leone in number of reported EVD cases, and make it the country with the highest mortality from EVD as of this date [3].

Outbreaks of EVD and other hemorrhagic fevers are associated with highly vulnerable populations in post-conflict countries with poorly performing economies and inadequate public health systems [4]. Previous outbreaks of EVD in Central Africa generally had fatality rates around 60–70% but sometimes as high as 90% [5]. Despite the somewhat lower mortality rate (58% in Liberia, based on case fatality of hospitalized patients), the recent epidemic was greater in magnitude because of vulnerable populations coping with other endemic diseases such as malaria, inadequate healthcare infrastructure, distrust of government workers, and spread to major population centers [2, 6, 7]. Cultural beliefs and practices, such as washing corpses before burial and touching at funerals, played an important role in transmission of EVD [2].

Post-conflict Liberia is one of the most socially-fragile countries on a continent likely to suffer adverse impacts of disease outbreaks such as EVD because of vulnerable social and natural systems [8], multiple interacting stressors, and low adaptive capacity [9]. Liberia was embroiled in a civil war between 1989 and 2003 [10], which destroyed much of its physical and social infrastructure. The war occurred in two phases as different groups gained control of the country, and the two conflicts produced >200,000 dead and displaced >700,000 Liberians [11]. Many of those displaced became refugees in neighboring countries (Guinea, 325,000; Côte d’Ivoire, 270,000; Sierra Leone, 125,000; Ghana, 8,000; Nigeria, 1,700), and an additional 500,000 were internally displaced in the capital Monrovia [12, 13]. The wars also produced a brain drain as skilled professionals, including most of the qualified doctors, sought refuge from the fighting in other countries [14]. Fighting was widespread, taking place in 10 of the 15 counties in Liberia [15]. To put current conditions in perspective, Liberia ranks 175 out of 186 countries on the latest Human Development Index, and among other disheartening ratings, boasts a Gross National Income per capita of US$752; 84% of the population is living on <US$1.25 per day [16].

Assessing social vulnerability to the potential effects of epidemics on people in Sub-Saharan Africa can be challenging because systematic information on socioeconomic conditions below the national or regional levels is often limited. The collapse of government institutions during the Liberian civil war exacerbated information scarcity; nevertheless, such assessments are needed to guide investments by government agencies and international donors in reducing social vulnerability to disease epidemics and other stressors and to strengthen adaptive capacity [17, 18]. Towards this end, we analyzed available data collected prior to the EVD outbreak [19] with an emphasis on visualizing spatial patterns of social vulnerability in rural Liberia. We developed a Social Vulnerability Classification using district and county level data from Liberia’s 2008 National Population and Housing Census (the first to be conducted in 24 years), and then mapped it to present a geo-referenced, relatively fine-grained view of relative social vulnerability among clusters of rural districts. The purpose of this article is to present the Social Vulnerability Classification, including methods; to assess its relevance for understanding social vulnerability to EVD in Liberia; and to offer it as a tool for estimating vulnerabilities that could lead to epidemics from future EVD and other disease outbreaks.

Vulnerability is defined generally as a measure of possible future harm [20]; methods and indicators for assessing vulnerability vary, depending on the research or policy context [21]. Efforts to use social indicators to construct indices characterizing social vulnerability at different spatial scales have proliferated over the past decade (e.g., [22–25] and studies reviewed in [26]). Most of these efforts address social vulnerability in the context of natural hazards and/or climate change, and are carried out in the United States or Europe, which are data rich. By contrast, our Social Vulnerability Classification was developed in the context of vulnerability to a disease outbreak, and is for a data-poor developing country in Africa. Furthermore, while our selection of census variables used to construct the vulnerability index is similar to the characteristics of vulnerability found in the social science literature (see Table 1 in [23]), they reflect the material reality of rural life in one of the world’s poorest countries. Our use of sub-county data better reflects the spatial variability of vulnerability but our data were insufficient to allow us to assess fully social capacity [24], which would have required more detailed household data (e.g., [27]). We believe our approach can be adopted elsewhere in situations that call for rapid, quantitative social vulnerability assessments at multiple scales in the context of disease outbreaks, demonstrating their relevance beyond the context of climate change and natural hazards.

## Methods and Materials

We selected 18 variables from the 2008 National Population and Housing Census conducted from 21–30 March 2008 by the Liberia Census Commission [19], and used them to construct a Social Vulnerability Classification. Technical assistance was provided to the Commission by the United Nations Population Fund to ensure that the census methodology met international standards. In Liberia, 15 counties and 135 districts at the sub-county level are delineated. Trained enumerators administered a standard questionnaire to households in 7,020 enumeration areas (EA). EAs were defined on the basis of clans in rural areas and communities in urban areas; each EA included 80–120 households. We used the most current EVD data available from the World Health Organization [3], recognizing that EVD cases are probably underreported, especially in rural areas [1]. We constructed our Social Vulnerability Classification at the district level because it was the scale most appropriate for examining potential relationships with readily available World Health Organization EVD data, which are only available at the county level.

The census variables we chose were those that served as potential indicators of the five dimensions of poverty [28]: (1) economic (income, ability to meet material needs); (2) human (health, education, nutrition); (3) political (rights, empowerment); (4) sociocultural (status, dignity, ability to participate as a valued member of society); and (5) protective (security, vulnerability). We acknowledge that vulnerability and poverty are not the same, but poverty (broadly defined) can serve as a proxy for vulnerability because the two are often highly correlated [29]. Poverty reduces people’s ability to cope with, recover from, or adapt to external stresses that affect their livelihoods and well-being, increasing their social vulnerability [18]. The 18 variables represent three of these dimensions of poverty (economic, human, and protective); it was difficult to find census data to adequately represent the political and sociocultural dimensions, but the five dimensions are often linked. We expressed the census variables as vulnerabilities. For example, one census variable was “% households producing fish”; we inverted that to “% households not producing fish”.

All statistics were performed using R version 3.1.2 R [30], a free software environment for statistical computing and graphics. The R package NbClust version 2.0.3 [31] was used for guiding the cluster analysis. The NbClust package provides 30 indices for determining the number of clusters and can propose to the user the best clustering scheme from the different results obtained by varying all combinations of number of clusters, distance measures, and clustering methods.

We used exploratory factor analysis with orthogonal rotation to investigate the underlying structure of the 18 variables. We expressed the 18 variables as a percentage of the population. An arcsine transformation is often used to improve the normality of the distribution of proportional data; however, when most values lie between 0.3 and 0.7, transformation is unnecessary [32], and the use of the transformation is demonstrably undesirable for a number of reasons (e.g., interpretability [33]). The arcsine transformation had little effect on the distributions of the 18 variables, and we chose not to transform the data.

An important decision in exploratory factor analysis is determining the number of factors to retain. We used principal components analysis to provide an estimate of the number of orthogonal components to retain for factor analysis; unfortunately, a wide range of criteria are available for assessing the number of non-trivial components, and most techniques suffer from an inherent subjectivity or tend to under- or overestimate the true dimension of the data [34]. Following [35], we examined a set of eight methods for determining the number of interpretable principal components. These methods included the Kaiser-Guttman, Joliffe’s modified Kaiser-Guttman, examination of the scree plot, the broken stick heuristic, parallel analysis, the number of components required to achieve 80% and 90% of variance explained, and the information dimension described by [35]. The number of components to be retained varied from a low of four to a maximum of twelve with an average of seven components (Table 1).

**Table 1:** Evaluation of the number of principal components to retain for factor analysis of 18 social vulnerability indicators using eight commonly used evaluation techniques.

Method	Principal Components Retained	References
Kaiser—Guttman [KG]	6	[36]
Jolliffe’s KG	8	[37]
Scree Plot	4 or 9	[34, 38]
Broken Stick	4	[34, 39, 40]
Parallel Analysis	4	[41–44]
80% Variance Explained	8	
90% of Variance Explained	12	
Information Dimension	11.9	[35]
Mean	7.43	

Determination of which variables contribute to each factor follows the methodology outlined by [45]. Rather than using an arbitrary threshold for the loadings, the threshold is set based on sample size by using the critical values for a simple correlation at α = 0.01 [two-tailed test] and doubling this value. For our data with 135 districts the critical value is approximately 0.222 which yields a threshold on the factor loadings of |0.444|. Using this threshold any variable identified as contributing to a factor will share at least 19% of its variance with the factor.

The overall social vulnerability of each district was classified through a cluster analysis of the seven factors identified above. The goal of the cluster analysis was to derive some broad characterization of social vulnerability to facilitate discussion and mapping. Clustering was performed using the k-means clustering algorithm [46]. The NbClust package [31] tested 25 metrics for k-means clustering with the number of clusters constrained to be between 2 and 7, and recommended 5 clusters based on majority rule among the available indices. Membership was well distributed among the 5 clusters as the smallest cluster contained 15 districts and largest 39 districts. All data were displayed spatially using ArcGIS 10.0 (ESRI, Redlands, CA). Data layers for county and district boundaries and roads were provided by the Liberia Institute of Statistics and Geo-Information Services (LISGIS).

## Results and Discussion

**Fig 1:**
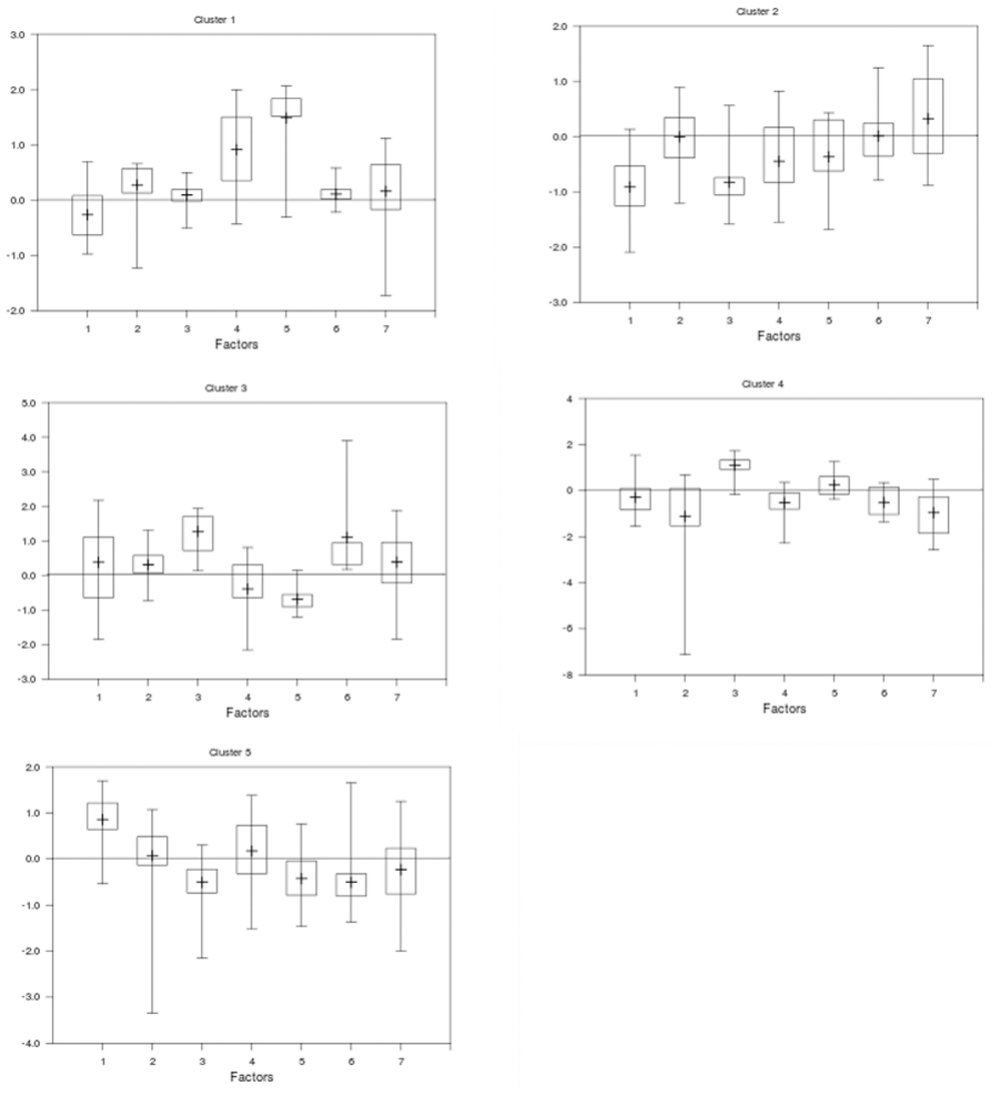
Distributions of district scores on seven factors. Distribution of social vulnerability scores from factor analysis for districts classified into five clusters (using NbClust) allowing evaluation of the influence of each respective social vulnerability factor on each cluster. For each cluster of districts, vertical lines indicate the mean (central cross bar) and maximum and minimum factor scores and boxes delineate quartile factor scores across all seven factors in each cluster of districts. Factor 1- Water Quality/Medical Proximity; Factor 2- Food Quality; Factor 3- Food Quantity; Factor 4- Displaced Populations; Factor 5 –Disabled and Dependent Populations; Factor 6 –Access to Land and Free Medical Care; Factor 7- Lack of Material Goods.

Exploratory factor analysis reduced our 18 variables (Table 2) to seven interpretable factors that explained 64% of the variance (Fig 1). The variance explained for the factor analysis is lower than the 77% produced by principal components analysis [38]; however, in the principal component analysis variables loaded onto multiple components, which reduces the explanatory power of the components. In the factor analysis, most variables loaded onto just one factor. Factor 1 is a “water quality-proximity to medical care” factor; Factor 2 relates to “food quality” and Factor 3 to “food quantity.” Factor 4 reflects the added stress on communities as “displaced populations,” and Factor 5 groups “disabled and dependent populations”. Factors 6 and 7 were not easily interpretable, but Factor 6 couples lack of access to free medical care and land; Factor 7 is most influenced by lack of material goods (furniture or mattresses). Loadings for most variables were associated with a single factor except for percentage of households lacking a mattress, which was associated with both displaced populations (Factor 4) and lack of material goods (Factor 7). We interpret this as instances of specific and general poverty. Factor 3 (food quantity) has an apparently contradictory positive correlation with undernourishment and negative correlation with stunted children. We speculate that in households that lack sufficient food, children do not survive to become stunted.

**Table 2:** Factors, factor loadings, and communalities for 18 social vulnerability attributes across Liberian districts as derived from exploratory factor analysis.

Census Variable (Dimension of Poverty)	Factors							Communality
	1	2	3	4	5	6	7	
% households without improved drinking water [E,H]	**0.891**		0.232		-0.205	-0.124	-0.114	0.922
% households that have unimproved waste disposal [H]	**0.855**		0.133					0.775
% households that are >80 minutes to a rural medical facility [H]	**0.702**						0.164	0.535
% households that are >20 minutes to drinking water [E]	**0.589**	0.152	-0.112	-0.181			-0.185	0.455
% population that is illiterate [H]	**0.575**			0.417	0.173		0.266	0.613
% households that do not produce livestock [E,H]		**0.858**	0.153	0.198	-0.193			0.844
% households that do not produce poultry [E,H]		**0.766**						0.603
% households that do not produce fish [E,H]		**0.729**	0.247			0.101		0.608
% undernourished [H]		-0.107	**0.802**			0.145	-0.166	0.712
% stunted children [H]			**-0.545**		-0.164		-0.154	0.354
% population that is displaced [PR]	-0.129		-0.122	**0.760**				0.621
% households without a mattress [E]			0.324	**0.630**	0.252		**0.595**	0.934
% population that is disabled [H, PR]	-0.220			0.354	**0.894**	0.130		0.995
% population that is dependent [PR]		-0.214	0.302	-0.107	**0.491**	-0.177	0.233	0.476
% households lacking access to free medical care, drugs, or both [H]			0.383	-0.116		**0.782**		0.789
% households without access to land [E]		0.135		0.101		**0.618**		0.426
% households without furniture [E]	0.159	0.427	-0.164	0.101		0.183	**0.540**	0.580
% households with substandard housing [E]	-0.124	0.255	-0.312	0.147		0.171	0.206	0.277
SS loadings	2.835	2.229	1.601	1.433	1.274	1.189	0.958	
Proportion of variance explained	0.157	0.124	0.089	0.080	0.071	0.066	0.053	
Cumulative variance explained	0.157	0.281	0.370	0.450	0.521	0.587	0.640	

Letters in () indicate the key dimension(s) of poverty reflected by the census variable. E = economic, H = human, PR = protective (the others are political and sociocultural). Entries in bold indicate loadings > 0.444. Goodness of fit of the model testing the null hypothesis of no difference between observed data and an hypothesized seven factor solution, χ^2^ = 56.57, df = 48; p-value 0.186.

**Fig 2:**
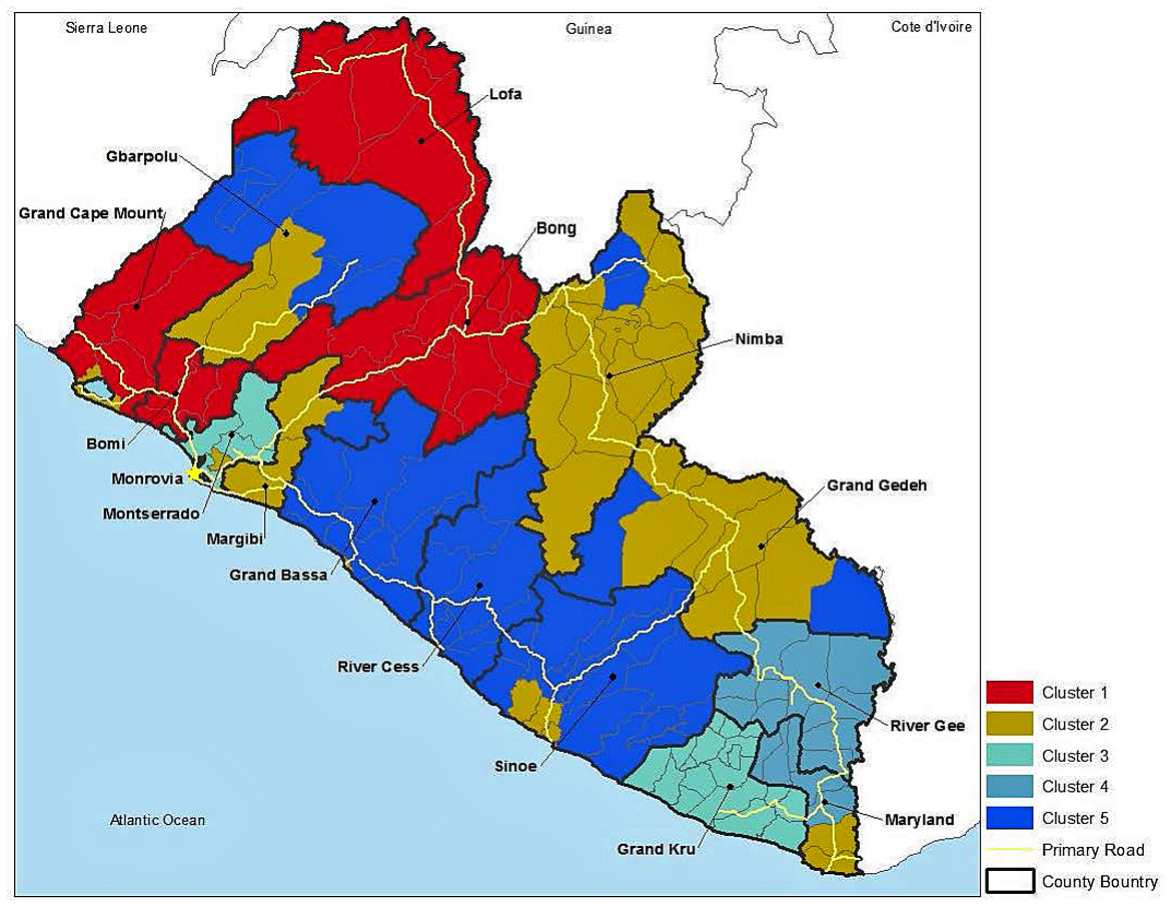
Clusters of social vulnerability in rural Liberia, by district. Based on strength and distribution of factor scores (see Fig 1), social vulnerability of each cluster of districts can be loosely ranked from most to least vulnerable as: Cluster 1, food quality, displaced persons, disabled, dependent populations; Cluster 3, food quantity, food quality, lack of access to land/free medical care; Cluster 4, food quantity, disabled dependent populations and Cluster 5, water quality/proximity to medical care; and finally, Cluster 2, no strong vulnerability scores (county boundaries are in black, district boundaries in gray, main roads in yellow).

Cluster analysis allowed classification of the districts based on their social vulnerability scores for each of the seven factors (Fig 2); some counties had districts in different clusters. Cluster 1 (Lofa, Bong, Grand Cape Mount, and Bomi Counties) showed the most overall vulnerability because it had the most positive scores among the seven factors with positive values for Factors 2 (food quality), 4 (displaced persons) and 5 (disabled and dependent populations). Cluster 3 (Montserrado and Grand Kru) was the next most vulnerable cluster, based on positive scores for food quantity (Factor 3), food quality, and lack of access to land/free medical care (Factor 6). Clusters 4 and 5 were somewhat vulnerable groups with differing concerns dominating. Cluster 4 (districts of River Gee County and northern Maryland County) had positive scores for food quantity (Factor 3) and disabled/dependent populations (Factor 5) with negative values for other factors. Water quality/proximity to medical facilities (Factor 1) was a concern for Cluster 5 (districts in Grand Bassa, River Cess, most of Sinoe and Gbarpolu, and portions of Margibi, Nimba, and Grand Gedeh Counties) and negative scores for food quantity (Factor 3), disabled and dependent populations (Factor 5), and access to land/free medical care (Factor 6). Cluster 2 (Nimba, Margibi, Grand Gedeh, some of Gbarpolu, and a few districts in Grand Cape Mount, Montserrado, Sinoe, and Maryland Counties) was the least vulnerable cluster with negative values for water quality/proximity to medical facilities (Factor 1) and food quantity (Factor 3).

We postulated that poor nutrition as well as lack of health services compound disease outbreak, and that infirmity and morbidity from disease outbreaks would contribute to a loss of productive labor and subsequently lower agricultural yields, creating greater food insecurity. Most rural Liberians (71.6%) live in severe poverty [47], and most rural households are food insecure, meaning that they lack access at all times of the year to sufficient, safe, and nutritious food to meet their food preferences and dietary needs for an active and healthy life [48]. Nationally, 81% of the rural population was either moderately vulnerable [41%] or highly vulnerable (40%) to either chronic or transitory food insecurity [48, 49]; these results are similar to our Factors 2 and 3 (Clusters 1, 3, and 4, and to a lesser extent Cluster 5). Food insecurity may be caused by lack of access to farmland, lack of income to purchase food or inputs such as improved seed or fertilizer, insufficient production including that caused by labor shortage, or inability to store food between harvests due to spoilage or other losses. Thus food scarcity and other economic impacts [50] likely will continue to cascade throughout the rural population long after the EVD outbreak is brought under control.

**Fig 3:**
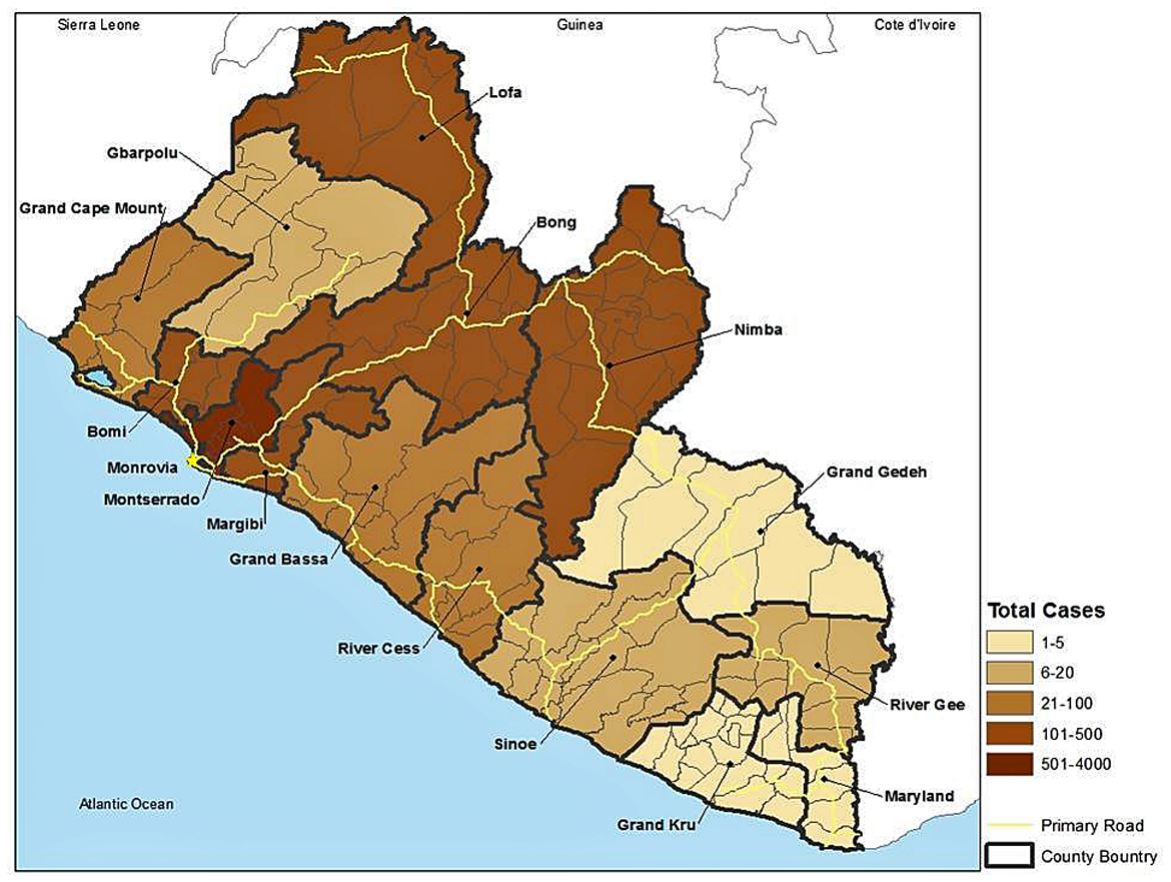
Geographical distribution of Ebola virus disease cases in Liberia, by county. Data are estimates made on 10 May 2015 [3]. Liberia had the second highest level of cumulative confirmed, probable, and suspected cases in West Africa as of this date, with the greatest mortality.

**Fig 4:**
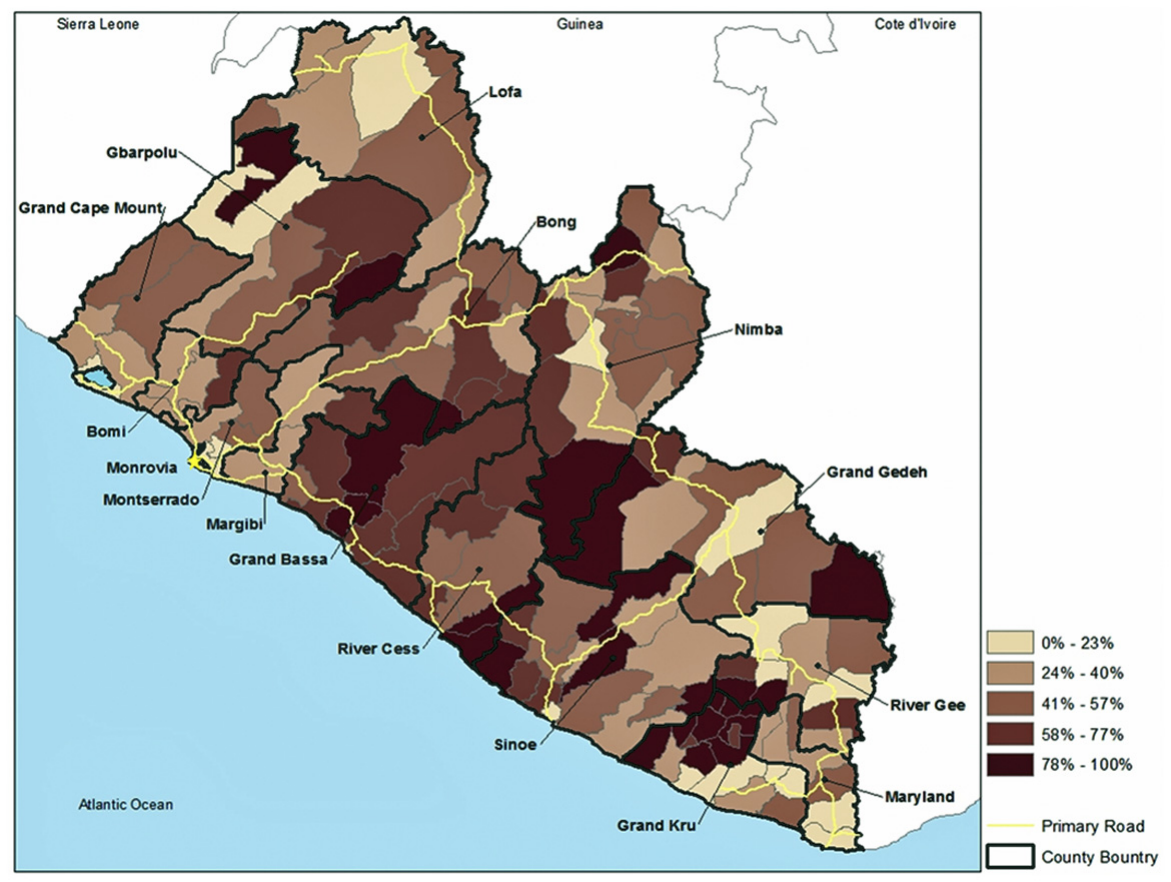
Percentage of households in each district that are more than 80 minutes travel to a healthcare facility. Travel by the rural population is primarily by foot, bicycle, motorbike, bush taxi, truck, or some combination. Organized public transportation is lacking and many roads are impassable in the rainy season.

The 2008 estimated population of Liberia was 3.673 million (estimated at 4.294 million in 2013) with about 58% living along the coast. The highest population density occurred in and around the capital Monrovia, including Montserrado and nearby Margibi and Bomi Counties. The most vulnerable rural districts (Cluster 1) have about 814,992 people. The populations of other vulnerable clusters were 121,480 (Cluster 4) and 464,683 (Cluster 5). Excluding Cluster 3, which was also a relatively vulnerable group and included slums around Monrovia, the three clusters of the most vulnerable districts contain 40% of the rural populace. Cultural factors and population density are important in aiding or hindering transmission of EVD and determining the number of victims. Nevertheless, comparing the locations of the reported cases of EVD (Fig 3) to our estimates of socially vulnerable populations (Fig 2) and proximity to medical facilities (Fig 4) illustrates the complex interactions of exposure and vulnerability in disease transmission and mortality.

EVD was introduced into Liberia in Lofa County and spread along main roads to the capital Monrovia (Montserrado County). The initial populations exposed to EVD were in the most vulnerable Cluster 1 in Lofa and Bong Counties, which suffered a relatively high occurrence of EVD. Similarly, Bomi County, also in Cluster 1, reported a high number of EVD cases. However, the highest numbers of infections were reported in the most densely populated Montserrado County that includes Monrovia. This county is comprised of districts that fall almost entirely into Cluster 3 (high social vulnerability).

More detailed analysis of EVD epidemiology will be required to link vulnerability to disease and disease transmission. The most likely reservoir species are bats [51] and primates (including humans), which are dead-end hosts [52]. Usually initial infection occurs by contact with the blood of another mammal through hunting or butchering [53]. Human-to-human transmission is by close physical contact with an infected person [7]. Those at highest risk are family members providing care, health care professionals, and people in contact with corpses [52]. But it is likely that the number of cases has been underreported, especially in rural areas; and, records may not reflect where victims were exposed. In addition, the World Health Organization data on EVD occurrence are reported at the county scale, but EVD cases tend to be clustered in local areas due to the nature of transmission. Moreover, county-scale EVD data are difficult to compare with social vulnerability classifications at the district scale. Finally, district-level assessment of social vulnerability masks variation in vulnerability that exists at the clan/community and household scales. Nevertheless, analyses such as ours point to “vulnerability hotspots” that deserve finer-scale assessment and attention.

Differential vulnerability among people at risk of exposure to EVD means that some people are more susceptible to its effects than others. For example, the lack of healthcare infrastructure in Liberia is acute. An assessment conducted in 2007 concluded that of the 389 functioning healthcare facilities, 300 were supported by NGOs. Some of those opened after the end of the civil war were scheduled to be closed [54]. Rural households mentioned the cost of medical care and distance to facilities as reasons for not seeking care [54]. Our analysis of census data puts this in spatial perspective; cost of medical care was important in Clusters 1 and 3, and most rural households must travel >80 minutes (by foot, motorbike, automobile/bus/truck or some combination), to reach medical care (Fig 4), especially districts in Cluster 5, where average time was estimated at >2.5 hours [54]. At the height of the epidemic, television news programs showed children and infants being carried in arms and adults in wheelbarrows, to medical facilities. All else being equal (e.g., age, other health factors)–good supportive medical care and early intervention are key to preventing mortality from EVD [2, 6].

Vulnerable populations and inadequate health and transportation infrastructure have contributed to the difficulty of controlling the EVD outbreak in West Africa [4, 6, 55]. Transportation networks are still in disrepair with parts of the country being largely inaccessible in the rainy season, further impeding relief efforts outside of the main cities and the rural populace from traveling to healthcare facilities. The importance of early detection, isolation of infected people, and protection of health care workers [6] can be clearly seen in the response of Firestone Liberia to the first case of EVD in the Firestone rubber tree plantation in Margibi County [56], where prompt corporate response and an existing healthcare and management infrastructure limited EVD transmission locally.

The dimensions of poverty that include rights and empowerment, sociocultural status, and security are important aspects of vulnerability but more difficult to assess using indicators assessed at the county or district scale; such variables are best assessed at the community level. The human dimension of poverty includes access to education. The conflict years in Liberia deprived an entire generation of the opportunity to develop human capacity [10] as most schools, universities, colleges, and health facilities were destroyed and a generation of young Liberians did not attend school [57]. The total collapse of the education system continues to deprive most young Liberians of the basic knowledge, skills, and resources needed to take advantage of the limited available employment opportunities [58]. The EVD outbreak, which caused schools throughout Liberia to close from July 2014 to February 2015, could have a similar long-term effect: another lost generation, caused directly by mortality and indirectly by child abandonment and stigmatization, as well as disruption of the school year and increased numbers of dropouts [59].

Despite the fact that civil violence has been quelled since 2003 and substantial international donor funding has flowed into Liberia (from governments, the United Nations, and NGOs), the effort to reconstruct the country has been largely unproductive and disappointing [57, 58]. Today water delivery systems, sanitation facilities and waste treatment, and centralized electricity are all but non-existent even in the largest city, Monrovia. Prior to the EVD outbreak, even operational medical facilities lacked potable water, lighting, equipment, and refrigeration [54].

The economic impact of EVD will be felt for some time [50]. For example, in neighboring Sierra Leone, which was destabilized by events in Liberia and suffered its own civil war [60], civil disruption caused lasting negative effects on firms and productivity, thereby impeding recovery [61]. Similar economic effects caused by the EVD outbreak are being felt in Liberia; for example, planned expansion in the mining sector was halted as contractors invoked *force majeure* and personnel left the country [62]. Although initial estimates of the economic impact of the EVD outbreak on West African economies were dire—based on worst-case scenarios and estimated to be as much as US$25 billion—more recent World Bank estimates are significantly lower [50]. The World Bank projects income losses in 2015 of US$1.6 billion in West Africa or 12% of the combined GDP of Guinea, Sierra Leone and Liberia (US$200 million in Liberia alone).

## Conclusions

The recent EVD epidemic in West Africa was unprecedented: EVD was unknown there before this outbreak, which was larger than previous outbreaks by an order of magnitude [2, 6]. Nevertheless, the symptoms, transmissibility, and mortality rates were typical of previous EVD outbreaks [2, 55]. Now that EVD is clearly established in West Africa, future outbreaks on the same or even larger scale are anticipated [4]. The improvements in healthcare infrastructure needed to respond to future outbreaks are obvious and pressing, but reducing the social vulnerability of rural populations is also needed to lower short-term morbidity and mortality and long-term cascading effects of future disruptions.

The approach to assessing and mapping social vulnerability represented by the Social Vulnerability Classification presented here has limitations, as do other social vulnerability index approaches [63]. Social vulnerability—whether to EVD, natural hazards, climate change, or other social and environmental threats—varies spatially [24]: between households in a community, from community to community, and between districts and administrative regions (e.g., counties). Our objective was to characterize vulnerability at the smallest scale practicable using the best available data while providing a national-scale assessment to highlight vulnerability “hot spots” in Liberia. At the appropriate scale, social vulnerability index approaches are useful for identifying people, regions, or sectors that are especially vulnerable [21]. But this type of assessment is insufficient for fully understanding the nature of social vulnerability locally. Instead, it points to places where more fine-scaled analysis is called for to inform decision-making about where and how to implement interventions to reduce vulnerability to future EVD outbreaks.

Another limitation is that vulnerability rankings and mapping at the district scale may mask variation at smaller scales; vulnerability is relative. The districts ranking lower on our vulnerability scale are not invulnerable to EVD; and some households and communities within the less vulnerable districts may be as vulnerable to EVD as those in high vulnerability districts. Not every community or household within a district having one color on the map has the same vulnerability. Furthermore, vulnerability changes over time, and we used data from only one time period (2008) for our classification. The lack of data between the previous Liberian census (1984) and 2008 limited the potential for meaningful analysis of temporal trends in vulnerability. Another limitation was our inability to capture some components of social vulnerability for the index. For example, we were unable to obtain any proxy measures for the sociocultural and political dimensions of poverty, which can play an important role in determining vulnerability to disease. Finally, the rigor of any Social Vulnerability Classification will be limited by the rigor of the data used to construct it. Although most indices have been built in countries having relatively rigorous, systematic, detailed social indicator data, such endeavors are also possible, and needed, in developing countries that lack rich data sets but nevertheless have sufficiently reliable data to work with.

Despite the limitations acknowledged here, we believe that our Social Vulnerability Classification and map are useful for drawing attention to districts of Liberia that are likely to have high social vulnerability to diseases like EVD. Mapping social vulnerability can help draw attention to its spatial patterns, and potential correlations between vulnerability and disease occurrence (or other phenomena) by facilitating integration with other types of data. The approach can be scaled up or down, depending on the scale of interest, assuming data are available at multiple spatial scales. Mapping vulnerability is an effective way of communicating information to decision-makers. And, it allows fairly rapid assessment to identify highly vulnerable places where in-depth, local-level investigation is warranted to evaluate where investments to reduce vulnerability and enhance adaptive capacity might be targeted. Thus, our Social Vulnerability Classification represents a first step in the process of better understanding the relationship between social vulnerability and disease in Liberia, and where specific improvements in livelihoods and living conditions may be most needed. More broadly, our methods and approach can be adopted elsewhere to guide efforts at reducing social vulnerability to disease epidemics, natural hazards, climate change, and other stressors.

## References

[1] Saéz AM, Weiss S, Nowak K, Lapeyre V, Zimmermann F, Düx A, et al Investigating the zoonotic origin of the West African Ebola epidemic. EMBOMM, 2015; 7: 17–23. 10.15252/emmm.201404792 PMC4309665 25550396

[2] World Health Organization Ebola Response Team. Ebola virus disease in West Africa—the first 9 months of the epidemic and forward projections. N Eng J Med 2014; 371: 1481–1495. 10.1056/NEJMoa1411100 PMC4235004 25244186

[3] World Health Organization. Ebola response roadmap situation report 13 May 2015 [internet]. Geneva: World Health Organization Available: http://apps.who.int/ebola/en/current-situation/ebola-situation-report-13-may-2015. Accessed 18 May 2015.

[4] Bausch DG, Schwarz L. Outbreak of Ebola virus disease in Guinea: Where ecology meets economy. PLoS Negl Trop Dis 2014; 8: e3056 Available: http://www.plosntds.org/article/info%3Adoi%2F10.1371%2Fjournal.pntd.0003056. Accessed 10 December 2014. 10.1371/journal.pntd.0003056 25079231 PMC4117598

[5] Feldmann H, Geisbert TW. Ebola haemorrhagic fever. Lancet 2011; 377: 849–862. 10.1016/S0140-6736(10)60667-8 21084112 PMC3406178

[6] MacNeil A, Rollin PE. Ebola and Marburg hemorrhagic fevers: neglected tropical diseases? PLoS Neg Trop Dis 2012; 6: e1546 Available: http://www.plosntds.org/article/info%3Adoi%2F10.1371%2Fjournal.pntd.0001546. Accessed 10 December 2014. 10.1371/journal.pntd.0001546 PMC3385614 22761967

[7] Fauci AS Ebola—underscoring the global disparities in health care resources. N Engl J Med 2014; 371(12):1084–6. 10.1056/NEJMp1409494 25119491

[8] Dixon RK, Smith J, Guill S. Life on the edge: Vulnerability and adaptation of African ecosystems to global climate change. Mitig Adapt Strateg Glob Change 2003; 8: 93–113.

[9] Boko M, Niang I, Nyong A, Vogel C. Chapter 9: Africa. Geneva: Intergovernmental Panel on Climate Change; 2007 Fourth Assessment Report of the IPCC.

[10] Hegre H, Østby G, Raleigh C. Poverty and civil war events: A disaggregated study of Liberia. J Conflict Resolut 2009; 53: 598–623.

[11] Ellis S. The Mask of Anarchy. 2nd ed New York: New York University Press; 2007.

[12] Bruce B. Liberia: the challenges of post-conflict reconstruction. Washington DC: Migration Information Source, Migration Policy Institute; 2004 Available: http://www.migrationpolicy.org/article/liberia-challenges-post-conflict-reconstruction. Accessed 10 December 2014.

[13] Kieh GK Jr. The roots of the second Liberian civil war. Int J World Peace 2009; 26: 7–30.

[14] Varpilah ST, Safer M, Frenkel E, Baba D, Massaquoi M, Barrow G. Rebuilding human resources for health: a case study from Liberia. Hum Resour Health. 2011; 9(1):11. 21569435 10.1186/1478-4491-9-11 PMC3117767

[15] Independent Evaluation Group. Liberia: World Bank Group country program evaluation, 2004, 2011 Approach Paper. Washington DC: World Bank; 2012 Available: http://documents.worldbank.org/curated/en/2011/12/15797447/liberia-world-bank-group-country-program-evaluation-2004-2011-approach-paper. Accessed 10 December 2014.

[16] United Nations. Human development report 2014. New York: United Nations Development Programme; 2014. Available: http://hdr.undp.org/en/countries/profiles/LBR. Accessed 10 December 2014.

[17] Brooks N, Adger WN, Kelly PM. The determinants of vulnerability and adaptive capacity at the national level and the implications for adaptation. Global Environ Change 2005; 15: 151–163.

[18] Kelly PM, Adger WN. Theory and practice in assessing vulnerability to climate change and facilitating adaptation. Climatic Change 2000; 47: 325–352.

[19] Government of Liberia. 2008 population and housing census final results. Monrovia: Liberia Institute of Statistics and Geo-Information Services; 2009.

[20] Wolf S, Hinkel J, Hallier M, Bisaro A, Lincke D, Ionescu C, et al Clarifying vulnerability definitions and assessments using formalisation. Int J Climate Change Strateg Manage. 2013; 5(1): 54–70.

[21] Hinkel J. Indicators of vulnerability and adaptive capacity: Towards a clarification of the science—policy interface. Glob Environ Change. 2011; 21(1): 198–208.

[22] Cutter SL, Finch C. Temporal and spatial changes in social vulnerability to natural hazards. Proc Nat Acad Sci. 2008; 105(7): 2301–6. 10.1073/pnas.0710375105 18268336 PMC2268131

[23] Cutter SL, Boruff BJ, Shirley WL. Social vulnerability to environmental hazards. Soc Sci Quart. 2003; 84(2): 242–61.

[24] Frazier TG, Thompson CM, Dezzani RJ. A framework for the development of the SERV model: A Spatially Explicit Resilience-Vulnerability model. Appl Geog. 2014; 51: 158–72.

[25] Abson DJ, Dougill AJ, Stringer LC. Using Principal Component Analysis for information-rich socio-ecological vulnerability mapping in Southern Africa. Appl Geog. 2012; 35(1): 515–24.

[26] Zebardast E. Constructing a social vulnerability index to earthquake hazards using a hybrid factor analysis and analytic network process (F’ANP) model. Nat Haz. 2013; 65(3): 1331–59.

[27] Hahn MB, Riederer AM, Foster SO. The Livelihood Vulnerability Index: A pragmatic approach to assessing risks from climate variability and change—A case study in Mozambique. Glob Environ Change. 2009; 19(1): 74–88.

[28] Organisation for Economic Co-operation and Development. The DAC Guidelines, Poverty Reduction. Paris: OECD Publications Service; 2001 Available: http://www.oecd.org/dac/povertyreduction/2672735.pdf. Accessed 10 December 2014.

[29] Wisner B, Blaikie P, Cannon T, Davis I. At Risk: Natural Hazards, People's Vulnerability, and Disasters. New York: Routledge; 2004.

[30] R Core Team. R: A language and environment for statistical computing. R Foundation for Statistical Computing, Vienna, Austria; 2014 Available: http://www.R-project.org/.

[31] Charrad M, Ghazzali N, Boiteau V, Niknafs A. NbClust: An R package for determining the relevant number of clusters in a data set. J Stat Software 2014; 61: 1–36.

[32] Sokal RR, Rohlf FJ. Biometry. 3rd ed New York: WH Freeman; 1995.

[33] Warton DI, Hui FKC. The arcsine is asinine: the analysis of proportions in ecology. Ecology 2011; 92: 3–10. 21560670 10.1890/10-0340.1

[34] Jackson DA. Stopping rules in principal component analysis: a comparison of heuristical and statistical approaches. Ecology 1993; 74: 2204–2214.

[35] Cangelosi R, Goriely A. Component retention in principal component analysis with application to cDNA microarray data. Biol Direct 2007; 2:2 Available: http://www.biology-direct.com/content/2/1/2. Accessed 12 December 2014. 17229320 10.1186/1745-6150-2-2 PMC1797006

[36] Bandalos DL, Boehm-Kaufman MR. Four common misconceptions in exploratory factor analysis In: Lance CE, Vandenberg RJ, editors. Statistical and Methodological Myths and Urban Legends. New York: Routledge; 2009 pp. 61–87.

[37] Jolliffe IT. Principal Component Analysis. 2nd Ed New York: Springer; 2002.

[38] Cattel RB. The scree test for the number of factors. Multivariate Behav Res 1966; 1: 245–76. 26828106 10.1207/s15327906mbr0102_10

[39] Bartkowiak A. How to reveal the dimensionality of the data. Appl Stoch Mod Data Anal 1991; 55: 64.

[40] Férre L. Selection of components in principal component analysis: a comparison of methods. Comput Statist Data Anal 1995; 19: 669–682.

[41] Horn JL. A rationale and test for the number of factors in factor analysis. Psychometrika 1965; 30: 179–185 14306381 10.1007/BF02289447

[42] Beauducel A. Problems with parallel analysis in data sets with oblique simple structure. Meth Psych Res 2001; 6:141–157.

[43] Peres-Neto PR, Jackson DA, Somers KM. How many principal components? Stopping rules for determining the number of non-trivial axes revisited. Comput Statist Data Anal 2005; 49: 974–990.

[44] Patil VH, Singh SN, Mishra S, Donavan DT. Efficient theory development and factor retention criteria: Abandon the ‘eigenvalue greater than one’ criterion. J Bus Res 2008; 61:162–170.

[45] Stevens JP. Applied Multivariate Statistics for the Social Sciences. 5th ed New York: Routledge/Taylor & Francis Group; 2009.

[46] Hartigan JA, Wong MA. A K-means clustering algorithm. Appl Stat 1979; 28: 100–108.

[47] Oxford Poverty and Human Development Initiative. Liberia country briefing, multidimensional poverty index data bank. Oxford: OPHI, Oxford University; 2014 [Accessed: 10 December 2014] Available: http://www.ophi.org.uk/multidimensional-poverty-index/mpi-2014/mpi-country-briefings/.

[48] Government of Liberia. Comprehensive assessment of the agricultural sector, Vol. 2.1 Monrovia: Ministry of Agriculture; 2007 Available: https://openknowledge.worldbank.org/bitstream/handle/10986/7674/431760SR0v20Wh110CAAS1LIB12111Feb08.txt?sequence=2. Accessed 10 December 2014.

[49] Government of Liberia. The state of food and nutrition insecurity in Liberia: Comprehensive food security and nutrition survey. Monrovia: Ministry of Agriculture; 2010 Available: http://home.wfp.org/stellent/groups/public/documents/ena/wfp231357.pdf. Accessed 10 December 2014.

[50] Thomas MR, Smith G, Ferreira FHG, Evans D, Maliszewska M, Cruz M, et al The economic impact of Ebola on sub-Saharan Africa: updated estimates for 2015. Washington, DC: World Bank Group; 2015 Available: http://documents.worldbank.org/curated/en/2015/01/23831803/economic-impact-ebola-sub-saharan-africa-updated-estimates-2015. Accessed 10 December 2014.

[51] Peterson AT, Papes M, Carroll DS, Leirs H, Johnson KM. Mammal taxa constituting potential coevolved reservoirs of filoviruses. J Mammal 2007; 88: 1544–1554.

[52] Pigott DM, Golding N, Mylne A, Huang Z, Henry AJ, Weiss DJ, et al Mapping the zoonotic niche of Ebola virus disease in Africa. *Elife* 2014; 3: e04395. 25201877 10.7554/eLife.04395 PMC4166725

[53] Pourrut X, Kumulungui B, Wittmann T, Moussavou G, Delicat A, Yaba P, et al The natural history of Ebola virus in Africa. Microbes Infect 2005; 7: 1005–1014. 16002313 10.1016/j.micinf.2005.04.006

[54] Tsimpo C, Wodon Q. Health in Liberia: Basic diagnostic using the 2007 CWIQ survey In Wodon Q, editor. Poverty and the Policy Response to the Economic Crisis in Liberia. Washington DC: World Bank; 2012 pp. 60–81.

[55] Meltzer MI, Atkins CY, Santibanez S, Knust B, Petersen BW, Ervin ED, et al Estimating the future number of cases in the Ebola epidemic—Liberia and Sierra Leone, 2014–2015. Morb Mortal Wkly Rep Surveill Summ 2014; 63: 1–14. Available: http://www.cdc.gov/mmwr/preview/mmwrhtml/su6303a1.htm?viewType=Print&viewClass=Print. Accessed 14 December 2014. 25254986

[56] Reaves EJ, Mabande LG, Thoroughman DA, Arwady MA, Montgomery JM. Control of Ebola Virus Disease—Firestone District, Liberia, 2014. Morb Mortal Wkly Rep 2014; 63: 959–965. Available: http://www.ncbi.nlm.nih.gov/pubmed/25340914. Accessed 10 December 2014. PMC5779472 25340914

[57] Independent Evaluation Group. Liberia country program evaluation: 2004–2011. Washington DC: World Bank; 2013 Available: http://documents.worldbank.org/curated/en/2012/07/17159969/liberia-country-program-evaluation-2004-2011-evaluation-world-bank-group-program. Accessed 10 December 2014.

[58] Government of Liberia. Liberia's vision for accelerating economic growth A development corridor growth study. Monrovia: Ministry of Planning and Economic Affairs; 2008.

[59] Johnson Sirleaf E. Ellen Johnson Sirleaf urges world help on Ebola. [internet] BBC NewsHour 19 Oct 2014. Available: http://www.bbc.com/news/world-africa-29680934. Accessed 10 December 2014.

[60] Salehyan I, Gleditsch KS. Refugees and the spread of civil war. Int Organ 2006; 60: 335–366.

[61] Collier P, Duponchel M. The economic legacy of civil war: Firm-level evidence from Sierra Leone. J Conflict Resolut 2013; 57: 65–88.

[62] ArcelorMittal. ArcelorMittal statement on operations in Liberia [08 Aug 2014] [internet]. Available: http://corporate.arcelormittal.com/news-and-media/press-releases/2014/aug/08-08-2014. Accessed 10 December 2014.

[63] Fekete A. Spatial disaster vulnerability and risk assessments: challenges in their quality and acceptance. Nat Haz. 2012; 61(3): 1161–78.

